# Accessory Antler Growing From the Left Zygomatic Bone of a Male Rusa Deer ( *Rusa timorensis* )

**DOI:** 10.1111/ahe.70129

**Published:** 2026-05-14

**Authors:** Uwe Kierdorf, Patrick Barrière

**Affiliations:** ^1^ Department of Biology University of Hildesheim Hildesheim Germany; ^2^ Agence néo‐Calédonienne de la Biodiversité – ANCB Kone New Caledonia

**Keywords:** antler abnormality, ectopic antler, New Caledonia, periosteum, trauma

## Abstract

This paper reports a case of accessory antler growth from the left zygomatic bone of a rusa stag (estimated age 7 years) shot in New Caledonia in January 2026. At the time of death, the individual was carrying orthotopic velvet antlers of about 13.5 cm length. The extra antler had grown from the lower rim of the left orbita and was covered by typical velvet. It was attached to the zygomatic bone via a bony socket that was covered by normal scalp skin and considered to represent a pedicle. The ectopic pedicle‐and‐antler structure (length 5.4 cm) became detached during maceration, suggesting that the connection between pedicle base and zygomatic bone had been cartilaginous rather than osseous. According to an observation of the stag's head shortly after death, a small exostosis was also present above the right eye. The distal portion of this exostosis appeared to be covered by velvet and thus may have represented a second accessory antler. However, since this region of the skull was destroyed by the shot in the head, it could not be examined after maceration. The case presented here is only the second instance of antler formation from the zygomatic bone reported in the scientific literature. Accessory antler growth was presumably caused by a trauma to the periosteum. The findings of the present study and other reports suggest that all membrane bones of the deer skull have the potential to produce accessory antlers upon strong unphysiological stimulation of their periosteum.

## 1 Introduction

Antlers are periodically replaced paired bony cranial appendages that are the most distinguishing trait of the family Cervidae (Goss 1983; Landete‐Castillejos et al. 2019; Heckeberg 2025). Except for the reindeer ( *Rangifer tarandus* ), antlers are normally grown only by males and thus constitute male secondary sexual characters (Goss 1983). Antlers do not develop directly from the skull roof, but are grown and cast from permanent bony projections of the frontal bones known as pedicles (Goss 1983; Kierdorf and Kierdorf 2002).

The onset of pedicle growth in young male deer is triggered by a temporary rise in circulating testosterone. When the pedicles have achieved a species‐specific threshold length, growth of the first antlers starts from their tips (Li and Suttie 1994; Kierdorf and Kierdorf 2002). It is with the casting of its first antlers that a male deer enters into the cycle of antler loss and regeneration. The mode of bone formation in antlers is a modified form of endochondral ossification (Banks and Newbrey 1983). Growing antlers are covered by a special type of integument, referred to as velvet, which differs in several aspects from normal scalp skin, including the presence of huge sebaceous glands and the lack of *arrector pili* muscles (Goss 1983).

The antler cycle of male deer is mediated by the variation of testosterone concentration in their blood (Bubenik 1990; Lincoln 1992; Suttie et al. 1995). During antler growth, testosterone levels are low. A steep rise in circulating testosterone at the end of the growth period causes full mineralization of the antlers and shedding of the velvet, which exposes the bare bony (hard) antlers. These are dead structures that remain firmly attached to the living pedicles as long as blood testosterone levels are high. A marked drop in circulating testosterone after the rut causes intense osteoclastic activity in the distal pedicles, followed by antler casting and, independently of casting, the onset of new antler growth (Goss 1983; Goss et al. 1992; Kierdorf and Kierdorf 1992; Lincoln 1992; Kierdorf et al. 2023).

The development of the primary cranial appendages, i.e., of pedicles and first antlers, in young deer depends on a specialized periosteum that overlies the prospective site of pedicle growth (the pedicle anlage area) of the frontal bones. Deletion of this so‐called antlerogenic periosteum (AP) prevents pedicle and antler growth, whereas its transplantation to other areas of the skull or even to postcranial sites causes ectopic pedicle and antler formation (Hartwig 1967; Hartwig and Schrudde 1974; Goss and Powel 1985; Kierdorf and Kierdorf 2000; Li and Suttie 2001). The capacity for ectopic antler formation demonstrates that the AP is capable of autonomous differentiation and committed to a specific differentiation pathway (Kierdorf and Kierdorf 2001; Li and Suttie 2001). Later studies showed that the AP contains neural crest‐derived mesenchymal stem cells that are ultimately responsible for pedicle and first antler formation (Wang et al. 2019; Ba et al. 2025). Annual antler regeneration is achieved by proliferation and differentiation of stem cells of the pedicle periosteum (PP) that are descendants of the AP stem cells (Kierdorf and Kierdorf 2001; Kierdorf et al. 2003; Li et al. 2007, 2010).

Naturally occurring accessory antlers have been observed in different deer species (Nitsche 1898; Brandt 1901; Finn 1903; Landois 1904; Seton 1909; von Raesfeld 1920, 1923; Dixon 1934; Wislocki 1952; Nellis 1965; Vandal et al. 1986; Bubenik and Hundertmark 2002; François 2011). The extra antlers are mostly located on the frontal bones and sometimes grow from the sides of the orthotopic pedicles. Size and shape of the accessory antlers vary widely, ranging from small knob‐like structures to larger outgrowths of relatively normal shape.

This paper describes a case of accessory antler formation from the left zygomatic bone of a male rusa deer ( *Rusa timorensis* ) shot in New Caledonia. The species was introduced to New Caledonia in 1870 as a diplomatic gift (Barrière and Fort 2021). New Caledonia is considered one of the most important current biodiversity hotspots (Myers 1988; Myers et al. 2000) and harbours the largest rusa deer population on Earth, estimated at 250,000–370,000 deer individuals, with local densities greater than 100 deer/km^2^ (Witczuk et al. 2025). The rusa deer is a major invasive alien species in New Caledonia and listed as a priority species in the territorial strategy for invasive alien species that threaten natural ecosystems (CEN Nouvelle‐Calédonie 2021). As a consequence, rusa deer can be hunted all‐year without limit in New Caledonia. Annually between 80,000 and 100,000 individuals are culled, and the species constitutes a major source of animal protein for the local human population. Males in velvet, mainly between January and April, are particularly appreciated because of their high fat content.

## 2 Case Presentation

On January 9, 2026, a rusa stag was shot by a hunter in Bas‐Farino (21.676930; 165.782620, municipality of Farino, New Caledonia) in the course of regular hunting operations under New Caledonian law. The animal was shot in the head with a 0.243 calibre rifle, which caused major damage to the skull, especially on the right side. According to the hunter, the stag was in good physical condition (dressed weight 68 kg) with no visible signs of old injuries, and its testicles appeared normal. The stag's head was collected by one of the authors (P.B.) 2 days after the animal had been shot, by which time decomposition under the hot and humid conditions of the rainy season had already begun. Thus, when the head was recovered, the velvet had peeled off from the antlers and some teeth had been lost. Based on mandibular tooth wear (CEN Nouvelle‐Calédonie 2009), the animal's age at death was estimated at 7 years.

The stag's head was prepared by anaerobic maceration. Following cleaning, drying and photographic documentation, the partly destroyed skull was reconstructed (P. B.) by gluing the recovered pieces into place and subsequent painting of the antlers. The specimen is currently held in the biological collection of the Agence néo‐Calédonienne de la Biodiversité in Koné, Presqu'ile de Foué, New Caledonia (collection number ANCB_4135).

**FIGURE 1:**
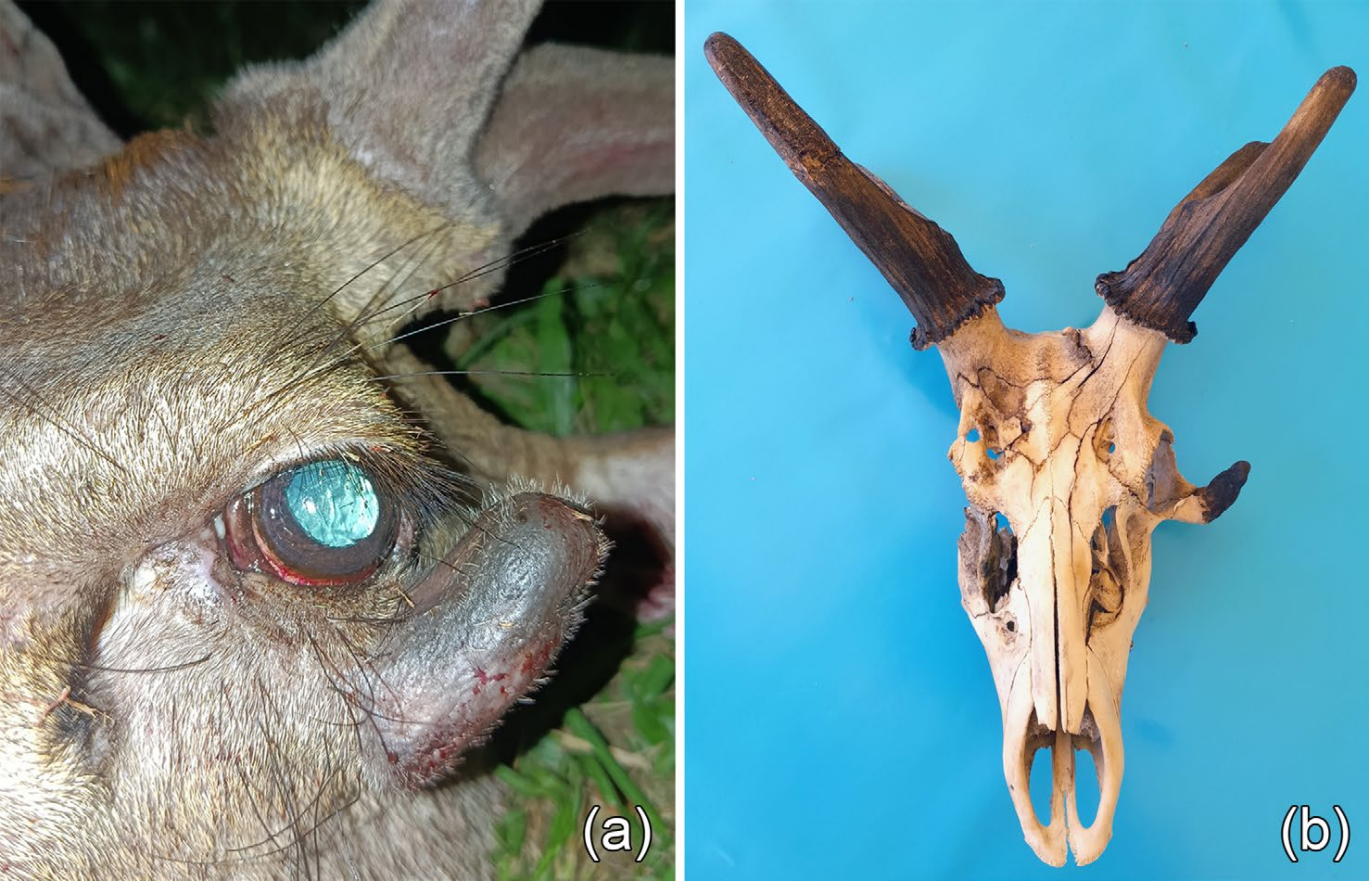
Accessory antler growth from the left zygomatic bone of the rusa stag. (a) Photo of the individual's head taken shortly after death. A velvet‐covered outgrowth emanates from below the left eye. The orthotopic antlers are also covered by velvet. (b) Dorsal view of the reconstructed dry skull (antlers painted), with the accessory pedicle and antler refitted to the lower rim of the left orbita. The length of the orthotopic antlers is about 13.5 cm.

When shot, the stag had grown normal velvet antlers of about 13.5 cm length, which corresponds to about 16 to 23% of the final antler length for 7 year‐old stags that ranges between approximately 60 and 85 cm. The orthotopic antlers are situated on pedicles of normal size and exhibit a symmetrical shape, with basal burrs (coronets), brow tines, and main beams (Figure 1a,b).

**FIGURE 2:**
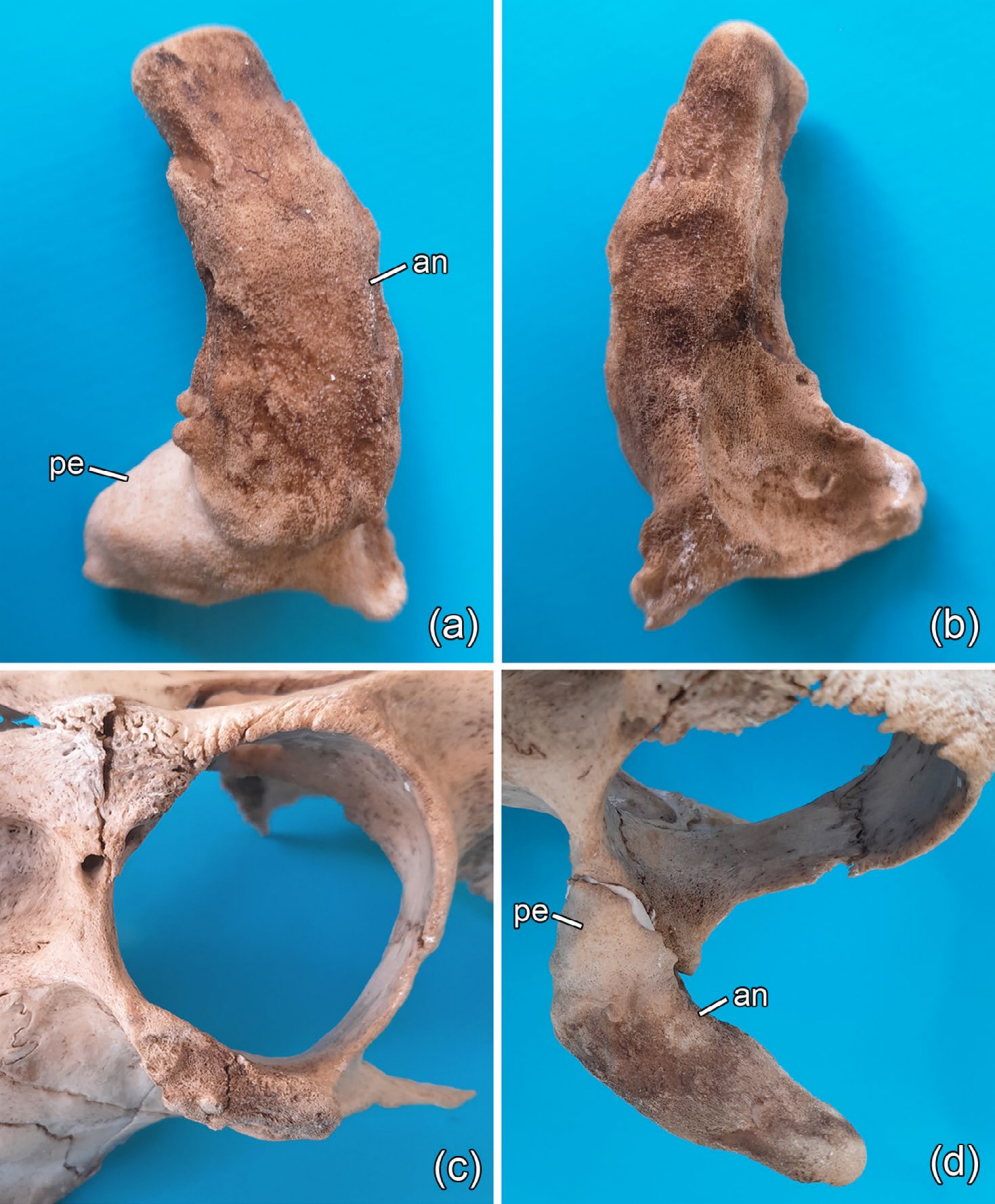
Accessory pedicle and antler structure and its attachment to the left zygomatic bone of the rusa stag. (a) Anterior view of the accessory outgrowth with pedicle portion (pe), which had been covered by normal scalp skin during life, and darker and more porous antler portion (an) that had been covered by velvet. (b) Posterior view of the accessory outgrowth. The basal concavity had probably been occupied by cartilage. (c) Left‐lateral view of the dry skull, showing uneven surface of the zygomatic bone at the site of attachment of the accessory pedicle‐and‐antler structure. (d) Dorsal view of the accessory pedicle (pe) and antler (an) structure refitted to the dry skull.

The most conspicuous feature of the stag's head was a bony outgrowth that had developed from below the left eye and grown in a lateral‐upward direction. As can be seen on the photo taken by the hunter shortly after the animal's death (Figure 1a), this outgrowth was covered by typical velvet, which proves its nature as an accessory antler. A burr is missing at the base of the ectopic antler, indicating that it is a first rather than a regenerated antler. The accessory antler was attached to the lower rim of the left orbita, formed by the zygomatic bone, via a bony socket that was covered by normal scalp skin. In the dried specimen, this basal socket, which is considered to represent a pedicle, is more lightly coloured and less porous than the antler itself (Figure 2a). The porous structure of the accessory antler is a typical feature of immature antlers that consist primarily of cancellous bone (Landete‐Castillejos et al. 2019). A concavity present on the posterior side of the antler (Figure 2b) might have been occupied by (unmineralized) cartilage that was lost during maceration, as was probably the case also with other cartilaginous portions of the accessory antler. The length of the ectopic pedicle‐and‐antler structure is 5.4 cm, its largest width at the base 1.9 cm.

In the course of the maceration process, the ectopic pedicle‐and‐antler structure became detached from the zygomatic bone, suggesting that the connection between pedicle base and underlying bone had been cartilaginous rather than osseous and was destroyed during maceration. At the site of attachment, the zygomatic bone is laterally broadened and exhibits an uneven surface structure (Figure 2c). There was no exact fit between pedicle base and zygomatic bone when the ectopic pedicle‐and‐antler structure was glued back into its original position (see Figure 1a) during reconstruction of the skull (Figure 2d). This is attributed to the destruction of the (supposed) interjacent cartilage layer during maceration.

According to the hunter who shot the stag, a small exostosis was also present above the animal's right eye. The distal portion of this outgrowth was supposedly covered by velvet. As this region of the cranium was not recovered due to the destruction caused by the shot in the head, it could not be examined in the dried specimen. Therefore, it is not possible to decide whether the exostosis represented another accessory antler structure.

**FIGURE 3:**
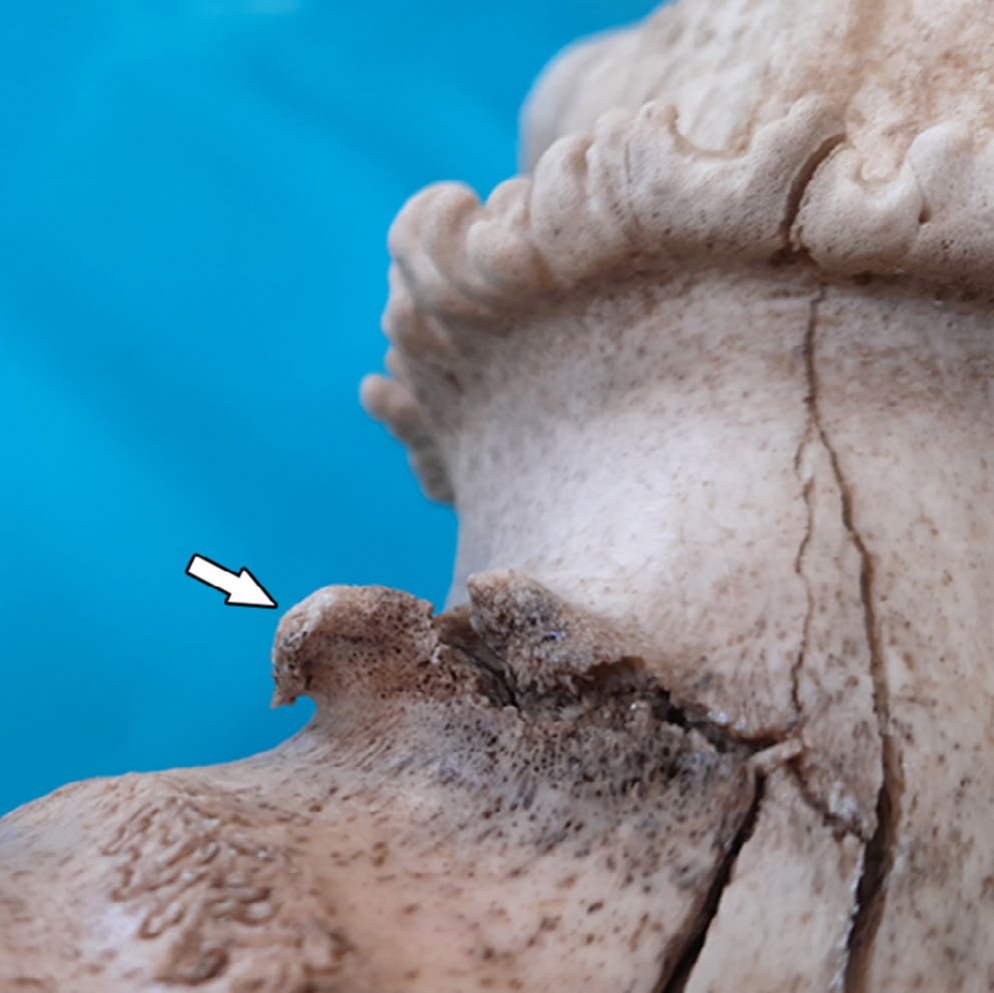
Osteophyte (arrow) located at the base of the left pedicle of the rusa stag (anterior view). A portion of the interfrontal suture is visible in the lower left corner of the image.

Additional findings in the macerated skull were (i) an osteophyte present on the medial site of the base of the left pedicle (Figure 3), (ii) signs of periodontal disease in the right maxilla, the condition having caused the loss of the right M^2^ and M^3^, and (iii) abnormal wear on the right M_3_.

## 3 Discussion

To the best of our knowledge, the case described here is only the second instance of accessory antler growth from the zygomatic bone reported in the scientific literature. Generally, development of accessory antlers from skull elements other than the frontal bones is a very rare phenomenon. A case of an ectopic pedicle plus antler (combined length 19.5 cm) located over the interparietal bone of a red deer stag ( *Cervus elaphus* ) was reported by Landois (1904). The specimen (a skull cap) also exhibited two orthotopic antlers of, respectively, 48 cm (right) and 49 cm (left) length. A burr at the base of the accessory antler indicated that it was a regenerated structure. According to Landois (1904), there was no bony connection between skull and ectopic pedicle, but the latter was held in position by the overlying skin. At the attachment site of the ectopic pedicle, the skull reportedly exhibited a penetrating hole that was diagnosed as a bullet wound by Landois (1904). If possible, a re‐examination of the specimen seems warranted. Accessory antler growth has also been described from the nasal bones of a mule deer ( *Odocoileus hemionus* ) and a white‐tailed deer (*Odocoileus virgininaus*) (Dixon 1934; Wislocki 1952).

**FIGURE 4:**
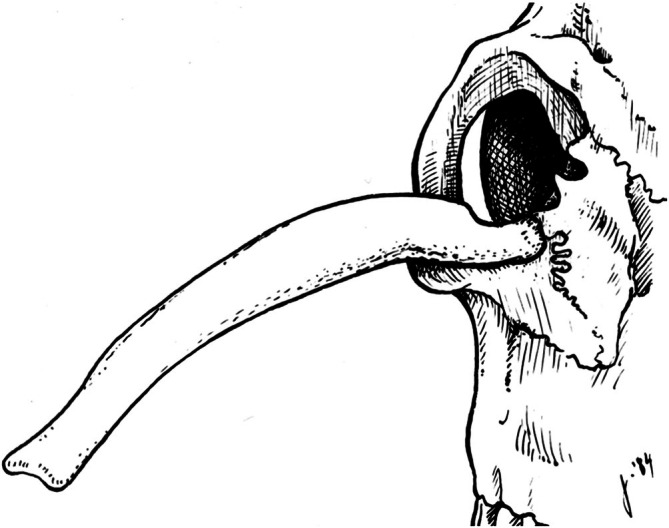
Accessory antler of 12.4 cm length that had developed from the right zygomatic bone of a white‐tailed deer buck. Drawing after photo of the reconstructed skull in Nellis (1965).

With regard to the current case, the most interesting previously reported specimen is an extra antler of 12.4 cm length that originated from the right zygomatic bone of an adult white‐tailed deer buck shot in the year 1962 in Montana, USA (Nellis 1965) (Figure 4). Like our rusa stag, the buck had been shot in the head and its skull was reconstructed. However, contrary to the present case, the buck was in hard antler when shot (right orthotopic antler with five, left orthotopic antler with four points). The accessory antler was likewise clean of velvet. According to Nellis (1965), the nature of the attachment of the accessory antler to the zygomatic bone indicated that antler replacement had never occurred at the site. From the report it remains unclear whether the accessory antler was attached to the zygomatic bone via a pedicle structure.

Each year, millions of male deer are killed and their antlered skulls carefully examined and collected as trophies by hunters. In consequence, there exists a vast body of literature on antler abnormalities. The fact that the case presented here is apparently only the second reported instance of accessory antler formation from the zygomatic bone therefore suggests that this is an extremely rare form of abnormality.

Regarding the cause of accessory antler formation in the rusa stag, a trauma to the periosteum of the zygomatic bone is considered the most likely explanation. This view is consistent with observations of accessory antler growth in captive deer following injuries to their skull (Bubenik and Hundertmark 2002). In the case described by Nellis (1965), the right eye of the white‐tailed deer was missing, which may have been the result of an injury that also triggered the growth of the accessory extra antler. An alternative explanation would be that the accessory antler in the rusa stag originated from developmentally displaced AP tissue. This is in principle conceivable, but seems less likely considering the “remote” location of the extra antler in the present case. Onset of extra antler growth from displaced AP tissue could have occurred later than the development of the orthotopic antlers. This is suggested by the observation that following transplantation of AP to the metacarpal region of a fallow buck ( *Dama dama* ) there was a lag period of several years before antler growth started from the initially formed ectopic pedicle (Kierdorf and Kierdorf 2000).

Replacement of accessory antlers, occurring (largely) in synchrony with that of the orthotopic antlers, has been observed (Dixon 1934; Bubenik and Hundertmark 2002), matching findings for ectopically formed antlers initiated by transplantation of AP (Hartwig and Schrudde 1974; Goss and Powel 1985). Interestingly, in the year following the death of the mule deer with an accessory antler situated on the nasal bones, Dixon (1934) observed another mule deer buck in the same locality that also exhibited an extra antler in its nasal region. Based on this observation, he hypothesized that the abnormality might have a hereditary basis. The currently available data do not allow any reliable conclusions regarding a potential genetic component in the development of accessory antlers, but this possibility should be kept in mind when discussing potential underlying causes. Unfortunately, the question of whether the rusa stag had grown a smaller accessory antler also on the right side of its head cannot be decided. If this had actually been the case, it would make the specimen even more extraordinary. The osteophyte present at the base of the stag's left pedicle is considered to be trauma‐induced.

Accessory antler growth subsequent to trauma indicates that the antler territory, i.e., the skull area capable of producing an antler, extends beyond the pedicle anlage area (Goss 1990; Kierdorf and Kierdorf 1992, 2002; Bubenik and Hundertmark 2002). Based on the location of extra antlers, Wislocki (1952, 75) had already concluded that “all of the membrane bones of the top of the skull of Cervidae appear to be endowed with a potential for antler growth, although the capacity appears to be much stronger in the frontals than in the regions anterior or posterior to them”. Activation of this growth potential requires a strong unphysiological stimulation of the periosteum, in most cases probably caused by trauma. In line with the view expressed by Wislocki (1952), it may be assumed that there exists a spatial variation in the responsiveness of the skull periosteum to unphysiological stimuli. This means that the intensity of the stimulus required for initiation of accessory antler formation may be higher at extra‐frontal skull sites than in the frontal bones. Accessory antler development from the orthotopic pedicles can most likely be attributed to traumatic stimulation of the PP. Naturally occurring ectopic antlers have not been reported from postcranial bones, indicating that their periosteum, unlike that of the skull's membrane bones, is incapable of initiating antler growth even when exposed to strong unphysiological stimuli.

In conclusion, accessory antler growth from the membrane bones of the deer skull demonstrates their potential for antler growth. Activation of this potential requires a strong unphysiological stimulation of the periosteum. In contrast, the determined periosteum (AP) overlying the prospective pedicle site is hormonally activated during puberty. It may be hypothesized that the responsiveness of the non‐antlerogenic skull periosteum to unphysiological stimulation varies over the antler cycle (Wislocki 1952; Bubenik and Hundertmark 2002). The present study demonstrates that the analysis of antler abnormalities like the one reported here can provide valuable insights into the mechanisms involved in antler development.

## Author Contributions

P.B. collected, prepared, measured, and photographed the specimen. U.K. and P.B. analysed the findings; U.K. drafted the original manuscript; U.K. and P.B. reviewed and edited the manuscript.

## Funding

The ANCB is mainly funded by the local authorities of New Caledonia (Provinces and Government) and the Rural Agency. No special funding was received for this study.

## Conflicts of Interest

The authors declare no conflicts of interest.

## Data Availability

The data that support the findings of this study are available from the corresponding author upon reasonable request.

## References

[ahe70129-bib-0001] Ba, H. , P. Hu , H. Yuan , et al. 2025. “RXFP2‐Positive Mesenchymal Stem Cells in the Antlerogenic Periosteum Contribute to Postnatal Development of Deer Antlers.” Communications Biology 8: 645. 10.1038/s42003-025-08085-w. 40263536 PMC12015367

[ahe70129-bib-0002] Banks, W. J. , and J. W. Newbrey . 1983. “Antler Development as a Unique Modification of Mammalian Endochondral Ossification.” In Antler Development in Cervidae, edited by R. D. Brown , 279–306. Caesar Kleberg Wildlife Research Institute.

[ahe70129-bib-0003] Barrière, P. , and C. Fort . 2021. “Monographie taxonomique sur le Cerf de Java *Rusa timorensis* .” In Atlas des mammifères sauvages de France. Volume 2: Ongulés et Lagomorphes, edited by A. Savouré‐Soubelet , C. Arthur , S. Aulagnier , et al., 96–101. Muséum national d'Histoire naturelle.

[ahe70129-bib-0004] Brandt, K. 1901. Das Gehörn und die Entstehung monströser Formen. Paul Parey.

[ahe70129-bib-0005] Bubenik, G. A. 1990. “Neuroendocrine Regulation of the Antler Cycle.” In Horns, Pronghorns, and Antlers, edited by G. A. Bubenik and A. B. Bubenik , 265–297. Springer.

[ahe70129-bib-0006] Bubenik, G. A. , and K. J. Hundertmark . 2002. “Accessory Antlers in Male Cervidae.” Zeitschrift für Jagdwissenschaft 48: 10–21. 10.1007/BF02285353.

[ahe70129-bib-0007] CEN Nouvelle‐Calédonie . 2009. “Comment estimer ou déterminer avec précision l'âge d'un cerf ? Schéma d'éruption et d'usure de référence de la naissance à 20 ans.” Conservatoire d'espaces naturels de Nouvelle‐Calédonie. https://www.ancb.nc/sites/default/files/st‐documents/st‐comment‐determiner‐l‐age‐d‐un‐cerf.pdf#page=2.

[ahe70129-bib-0008] CEN Nouvelle‐Calédonie . 2021. “New Caledonia's Strategy for Invasive Alien Species That Threaten Natural Ecosystems. Summary and list of the 105 main IAS and of the 68 priorities.” 2nd ed. (pp. 1−12). https://www.ancb.nc/sites/default/files/st‐documents/cen‐2021‐nc‐strategy‐for‐ias‐bd.pdf.

[ahe70129-bib-0009] Dixon, J. S. 1934. “A Study of the Life History and Food Habits of Mule Deer in California. Part I – Life History.” California Fish & Game 20: 181–282.

[ahe70129-bib-0010] Finn, F. 1903. “Exhibition of Drawings of, and Remarks Upon, An Abnormal Pair of Horns of the Barking‐Deer (*Cervulus muntjac*).” Proceedings of the General Meetings for Scientific Business of the Zoological Society of London II: 2–3.

[ahe70129-bib-0011] François, A. 2011. Abnorme Rehböcke. Bizarre Gehörne und ihre Ursachen. BLV Buchverlag.

[ahe70129-bib-0012] Goss, R. J. 1983. Deer Antlers: Regeneration, Function, and Evolution. Academic Press.

[ahe70129-bib-0013] Goss, R. J. 1990. “Of Antlers and Embryos.” In Horns, Pronghorns, and Antlers, edited by G. A. Bubenik and A. B. Bubenik , 298–312. Springer.

[ahe70129-bib-0014] Goss, R. J. , and R. S. Powel . 1985. “Induction of Deer Antlers by Transplanted Periosteum I. Graft Size and Shape.” Journal of Experimental Zoology 235: 359–373. 10.1002/jez.1402350307. 4056697

[ahe70129-bib-0015] Goss, R. J. , A. Van Praagh , and P. Brewer . 1992. “The Mechanism of Antler Casting in the Fallow Deer.” Journal of Experimental Zoology 264: 429–436. 10.1002/jez.1402640408. 1460440

[ahe70129-bib-0016] Hartwig, H. 1967. “Experimentelle Untersuchungen zur Entwicklungsphysiologie der Stangenbildung beim Reh (*Capreolus c. capreolus* L. 1758).” Roux’ Archiv für Entwicklungsmechanik 158: 358–384. 10.1007/BF01380537. 28304504

[ahe70129-bib-0017] Hartwig, H. , and J. Schrudde . 1974. “Experimentelle Untersuchungen zur Bildung der primären Stirnauswüchse beim Reh ( *Capreolus capreolus* L.).” Zeitschrift für Jagdwissenschaft 20: 1–13. 10.1007/BF01901843.

[ahe70129-bib-0018] Heckeberg, N. S. 2025. “The Systematics of Cervidae.” In Deer of the World: Ecology, Conservation and Management, edited by M. Melletti and S. Focardi , 3–44. Springer Nature.

[ahe70129-bib-0019] Kierdorf, H. , and U. Kierdorf . 1992. “State of Determination of the Antlerogenic Tissues With Special Reference to Double‐Head Formation.” In The Biology of Deer, edited by R. D. Brown , 525–531. Springer.

[ahe70129-bib-0020] Kierdorf, H. , and U. Kierdorf . 2001. “The Role of the Antlerogenic Periosteum for Pedicle and Antler Formation in Deer.” In Antler Science and Product Technology, edited by J. S. Sim , H. H. Sunwoo , R. J. Hudson , and B. T. Jeon , 33–51. Antler Science and Product Technology Research Centre.

[ahe70129-bib-0021] Kierdorf, U. , S. Gomez , S. R. Stock , O. Antipova , and H. Kierdorf . 2023. “Bone Resorption and Formation in the Pedicles of European Roe Deer ( *Capreolus capreolus* ) in Relation to the Antler Cycle—A Morphological and Microanalytical Study.” Journal of Anatomy 243: 842–859. 10.1111/joa.13908. 37278321 PMC10557394

[ahe70129-bib-0022] Kierdorf, U. , and H. Kierdorf . 2000. “Delayed Ectopic Antler Growth and Formation of a Double‐Head Antler in the Metacarpal Region of a Fallow Buck ( *Dama dama* L.) Following Transplantation of Antlerogenic Periosteum.” Annals of Anatomy 182: 365–370. 10.1016/S0940-9602(00)80013-8. 10932327

[ahe70129-bib-0023] Kierdorf, U. , and H. Kierdorf . 2002. “Pedicle and First Antler Formation in Deer: Anatomical, Histological, and Developmental Aspects.” Zeitschrift für Jagdwissenschaft 48: 22–34. 10.1007/BF02285354.

[ahe70129-bib-0024] Kierdorf, U. , E. Stoffels , D. Stoffels , H. Kierdorf , T. Szuwart , and G. Clemen . 2003. “Histological Studies of Bone Formation During Pedicle Restoration and Early Antler Regeneration in Roe Deer and Fallow Deer.” Anatomical Record 273A: 741–751. 10.1002/ar.a.10082. 12845710

[ahe70129-bib-0025] Landete‐Castillejos, T. , H. Kierdorf , S. Gomez , et al. 2019. “Antlers—Evolution, Development, Structure, Composition, and Biomechanics of an Outstanding Type of Bone.” Bone 128: 115046. 10.1016/j.bone.2019.115046. 31446115

[ahe70129-bib-0026] Landois, H. 1904. “Eine dritte Edelhirsch‐Geweihstange über dem mit der Hinterhauptsschuppe verwachsenen Zwischenscheitelbein.” Archiv für Entwicklungsmechanik der Organismen 18: 289–295. 10.1007/BF02163660.

[ahe70129-bib-0027] Li, C. , C. G. Mackintosh , S. K. Martin , and D. E. Clark . 2007. “Identification of Key Tissue Type for Antler Regeneration Through Pedicle Periosteum Deletion.” Cell and Tissue Research 328: 65–75. 10.1007/s00441-006-0333-y. 17120051

[ahe70129-bib-0028] Li, C. , and J. M. Suttie . 1994. “Light Microscopic Studies of Pedicle and Early First Antler Development in Red Deer ( *Cervus elaphus* ).” Anatomical Record 239: 198–215. 10.1002/ar.1092390211. 8059982

[ahe70129-bib-0029] Li, C. , and J. M. Suttie . 2001. “Deer Antlerogenic Periosteum: A Piece of Postnatally Retained Embryonic Tissue?” Anatomy and Embryology 204: 375–388. 10.1007/S004290100204. 11789985

[ahe70129-bib-0030] Li, C. , F. Yang , S. Haines , et al. 2010. “Stem Cells Responsible for Deer Antler Regeneration Are Unable to Recapitulate the Process of First Antler Development—Revealed Through Intradermal and Subcutaneous Tissue Transplantation.” Journal of Experimental Zoology Part B 314B: 552–570. 10.1002/jez.b.21361. 20549758

[ahe70129-bib-0031] Lincoln, G. A. 1992. “Biology of Antlers.” Journal of Zoology 226: 517–528. 10.1111/j.1469-7998.1992.tb07495.x.

[ahe70129-bib-0032] Myers, N. 1988. “Threatened Biotas: “Hot Spots” in Tropical Forests.” Environmentalist 8: 187–208. 10.1007/BF02240252. 12322582

[ahe70129-bib-0033] Myers, N. , R. A. Mittermeier , C. G. Mittermeier , G. A. B. da Fonseca , and J. Kent . 2000. “Biodiversity Hotspots for Conservation Priorities.” Nature 403: 853–858. 10.1038/35002501. 10706275

[ahe70129-bib-0034] Nellis, C. H. 1965. “Antler From Right Zygomatic Arch of White‐Tailed Deer.” Journal of Mammalogy 46: 108–109. 10.2307/1377829.

[ahe70129-bib-0035] Nitsche, H. 1898. Studien über Hirsche (Gattung Cervus im weitesten Sinne). Heft I. Untersuchungen über mehrstangige Geweihe und die Morphologie der Hufthierhörner im Allgemeinen. Wilhelm Engelmann.

[ahe70129-bib-0036] Seton, E. T. 1909. Life‐Histories of Northern Animals. An Account of the Mammals of Manitoba. Vol I. Grass‐Eaters. Charles Scribner's Sons.

[ahe70129-bib-0037] Suttie, J. M. , P. F. Fennessy , K. R. Lapwood , and I. D. Corson . 1995. “Role of Steroids in Antler Growth of Red Deer Stags.” Journal of Experimental Zoology 271: 120–130. 10.1002/jez.1402710207. 7884386

[ahe70129-bib-0038] Vandal, D. , C. Barrette , and H. Jolicoeur . 1986. “An Ectopic Antler in a Male Woodland Caribou ( *Rangifer tarandus caribou* ) in Québec.” Zeitschrift für Säugetierkunde 31: 52–54.

[ahe70129-bib-0039] von Raesfeld, F. 1920. Das Rotwild. 3rd ed. Paul Parey.

[ahe70129-bib-0040] von Raesfeld, F. 1923. Das Rehwild. 3rd ed. Paul Parey.

[ahe70129-bib-0041] Wang, D. , D. Berg , H. Ba , H. Sun , Z. Wang , and C. Li . 2019. “Deer Antler Stem Cells Are a Novel Type of Cells That Sustain Full Regeneration of a Mammalian Organ – Deer Antler.” Cell Death and Disease 10: 443. 10.1038/s41419-019-1686-y. 31165741 PMC6549167

[ahe70129-bib-0042] Wislocki, G. B. 1952. “A Possible Antler Rudiment on the Nasal Bones of a Whitetail Deer ( *Odocoileus virginianus borealis* ).” Journal of Mammalogy 33: 73–76. 10.2307/1375644.

[ahe70129-bib-0043] Witczuk, J. , S. Pagacz , and R. Alliod . 2025. “Estimating Relative Density of an Invasive Ungulate in a Biodiversity Hotspot Using Drone‐Based Thermal Video Surveys.” NeoBiota 103: 85–106. 10.3897/neobiota.103.157791.

